# Capsaicin regulates lipid metabolism through modulation of bile acid/gut microbiota metabolism in high-fat-fed SD rats

**DOI:** 10.29219/fnr.v66.8289

**Published:** 2022-05-26

**Authors:** Ting Gong, Haizhu Wang, Shanli Liu, Min Zhang, Yong Xie, Xiong Liu

**Affiliations:** 1 College of Food Science, Southwest University, Chongqing, People’s Republic of China; 2 Chongqing Medical and Pharmaceutical College, Chongqing, People’s Republic of China

**Keywords:** capsaicin, bile acid, gut microbiota, metabolism, high-fat diet

## Abstract

Capsaicin (CAP) is one of the active ingredients found in chili peppers and has been shown to reduce fat. This study aimed to explore the mechanisms of CAP activity by investigating intestinal microorganisms and bile acids (BAs). This study utilized 16S RNA sequencing to detect gut microbiota in cecal contents, and BAs in Sprague Dawley (SD) rats were also investigated. The results showed that 1) CAP increased the levels of chenodeoxycholic acid (CDCA), deoxycholic acid (DCA), β-muricholic acid (β-MCA), and tauro-β-muricholic acid sodium salt (T-β-MCA), which can regulate farnesoid X receptor (FXR) to inhibit Fgf15, increased CYP7A1 expression to lower triglycerides (TG) and total cholesterol (TC); 2) CAP decreased the abundance of *Firmicutes* and promoted the presence of specific fermentative bacterial populations, like *Akkermansia*; meanwhile, less optimal dose can reduce *Desulfovibrio*; 3) CAP decreased inflammatory factors IL-6 and IL-1β, and increased transient receptor potential channel of vanilloid subtype 1 (TRPV1) to regulate lipid metabolism, fasting plasma glucose and insulin resistance. In conclusion, CAP can reduce fat accumulation by regulating BAs, microorganisms, and short-chain fatty acids.

**Figure:**
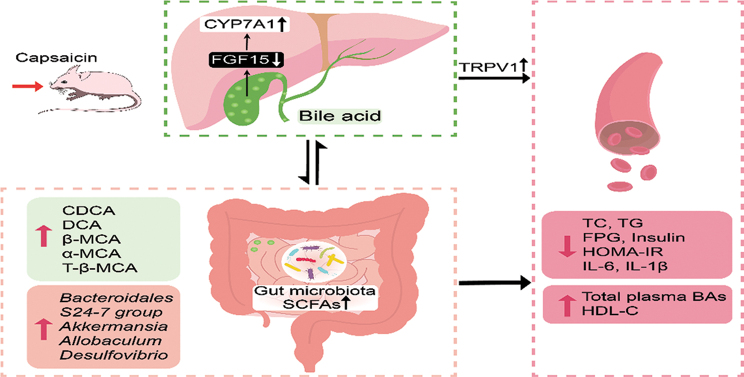

## Popular scientific summary

- CAP change the composition of bile acid to regulated lipid metabolism.
- CAP stimulates the secretion of SCFA by regulating gut microbiota.

The human intestine contains a large number of commensal microorganisms, collectively known as ‘intestinal microbiota’ that are essential for maintaining the integrity and function of the mucosal barrier, absorbing of nutrients, and maintaining energy homeostasis (1). Therefore, gut microbiota is now accepted as an integral part of the metabolome in a ‘super-organismal’ context (2). Recent innovations and developments in metabolomics and metagenomics have promoted the discovery of many microbe-derived small molecules and related genes, helping to decode metabolic host–microbe interactions (3). A recent research study has identified many metabolic characteristics of the symbiotic relationship between gut microbiota and the host, which provides useful information for understanding of human health (4).

Abnormalities in lipid metabolism are associated with metabolic syndromes (5). The development of metabolic syndrome in the human body is associated with altered microbial composition (6), suggesting that gut microbiota is a potential target for the prevention and treatment of metabolic syndrome (7). This idea has attracted widespread attention in recent years (8), and numerous natural food materials capable of regulating gut microbes have been studied as potential agents for the prevention and/or treatment of obesity (9).

Capsaicin (CAP, *trans*-8-methyl-N-vanillyl-6-nonenamide), one of the active ingredients in chili peppers, is a member of the vanilloid family of compounds (e.g. vanillin from vanilla, eugenol from bay leaves, cloves, and zingerone from ginger) (10). CAP has been found to be able to ameliorate lipid metabolism imbalance in rodents and other species. However, the mechanism underlying this activity is not yet fully understood. Some studies have suggested potential molecular mechanisms: 1) the presence of several single rotatable bonds in the CAP chemical skeleton can make the switching from reversible open-close conformations of capsaicin possible (10). CAP binds to a transient receptor potential channel of vanilloid subtype 1 (TRPV1) found on A- and C-delta fibers in the nociceptive sensory pathway, which initiates the signal transduction cascade that finally leads to desensitization of the afferent nerve fibers (11, 12). 2) The Cu (II)-chelating ability of CAP was the first aspect investigated in the exploration of the potentiality of this compound as an OIL antioxidant, which prevented the radical from reaching biomolecules, such as DNA, proteins, and lipids (10). However, Yuanwei Wang et al. reported that the reduced food intake and lipid metabolism regulation observed following CAP treatment were primarily mediated by changes in gut microbiota populations and concentrations of short-chain fatty acid (SCFA) (13). Other studies further emphasized the role of gut microbes in fat control by CAP. For example, Baboota et al. showed that oral administration of CAP led to weight loss in high-fat diet (HFD)-fed mice by inducing changes in gut microbiota compositions (14). Both Shen et al. and Kang et al. have also confirmed the effects of CAP on gut microbiota in HFD-fed mice by high-throughput sequencing (15, 16). Their research revealed that the majority of bacteria detected in fecal samples from healthy human volunteers belonged to two phyla, *Bacteroidetes* and *Firmicutes*. In addition, changes in predominant gut microbes were linked to excessive body fat, as evidenced in both humans and mice, with obese individuals characterized by more *Firmicutes* and fewer *Bacteroidetes* compared with lean individuals (17). However, the majority of these studies were performed based on the gavage that cannot adequately reflect the true situation. We also know that the CAP is absorbed by a nonactive process from the stomach and whole intestine, where the total absorption capacity varies between 50 and 90% in different animal studies (18–20). Thus, we chose oral administration for the experiment. Meanwhile, repeated application of high doses of CAP could lead to desensitization (21), so we need to consider whether the same effect can be achieved at lower doses.

This research work aimed to 1) explore the mechanism by which CAP regulates lipid metabolism through bile acid (BA) and gut microbiota; 2) identify the metabolic and taxonomic targets of bacteria, which can be used to treat and/or diagnose obesity; 3) elucidate whether less than optimal dose (0.005% food weight) can also improve abnormalities of lipid metabolism. This study is expected to provide a potential strategy to improve host health by regulating the gut microbiota using CAP as a promising example.

## Materials and methods

### Materials

CAP (≥95%) was purchased from Henan bis-biotech Co., Ltd, China.

### Animals and treatments

Animal experiments were conducted using male Sprague-Dawley (SD) rats obtained from Hunan SJA Laboratory Animal Co., Ltd, China, at 4–5weeks of age and used at 6–7 weeks of age. All experimental protocols were approved by the Institutional Animal Care and Use Committee of Southwest University (IACUC-20201008 01). All procedures were performed in strict accordance with EU Directive 2010/63/EU for animal experiments. Rats (200 ± 20 g) were housed in individual stainless steel cages at 25 ± 1°C, with relative humidity of 40–70% under a 12-h/12-h light/dark cycle. The experiment lasted for 4 weeks. Before the experiment, rats were provided free access to water and diet for a week. Rats were randomly divided into four groups (*n* = 8): control (low-fat) group (NC), HFD group, HFD with an optimal dose (0.01%) of CAP (HFM) (15, 22), and HFD with lower-than-optimal dose (0.005%) of CAP (HFL). The ingredient compositions of experimental group diets are shown in Supplementary Table 1. High-cholesterol feed contained 1% cholesterol supplement based on the US Standard AIN-93 feed formula (23).

### Sample collection

Animal mortality was monitored once a day throughout the trial duration. Body weight and food consumption were recorded once a week. After 4 weeks, SD rats were euthanized to collect blood, liver, cecal content, and small intestine content. Whole blood samples are taken by venipuncture. Following centrifugation, the supernatant was removed. Other samples were placed in sterile doffer tubes and immediately frozen in liquid nitrogen for 30 sec, after which they were stored at −80°C until further use. All operations were performed under aseptic conditions.

### Biochemical analysis

Plasma triglyceride (TG) and total cholesterol (TC) concentrations were determined using commercially available kits (BioSino, Beijing, China). The levels of fasting plasma glucose (FPG), high-density lipoprotein (HDL-C), and low-density lipoprotein (LDL-C) were measured using an automatic biochemical analyzer (URIT-8021A). The concentration of plasma fasting insulin was measured using a Rat Insulin (INS) ELISA kit (Sinobestio, Shanghai), which was purchased from Nanjing Jiancheng Bioengineering Institute (Nanjing, China). ELISA was also performed to assess serum inflammation-related factors, including IL-1β and IL-6.. The total BAs size in serum was measured with total BAs kit, and plasma Fgf15 was measured using the enzyme-linked immunoassay (ELISA) kit.

Hepatic lipid extraction and measurement: Lipid was extracted from frozen liver samples by chloroform: methanol using an adaptation of the Folch method (24). In short, 50 mg liver samples were homogenized using TissueLyzer (60 Hz, 30 sec) in chloroform and methanol mixture (2:1) followed by addition of methanol, chloroform, and ultrapure water. Extracted lipids were dried with N_2_ gas. After evaporation of organic solvents, the total extracted lipid was weighed and the lipid content of liver was calculated.

### Quantification of SCFAs by gas chromatography

Levels of SCFAs in cecal contents were determined according to the method described by Zhang Lei et al. (25). Cecal contents (0.5 mg) were weighed and dissolved in 2 mL of a mixture containing crotonic acid (5 mM) and sodium hydroxide (10 mM). The supernatants were collected after centrifugation at 10,000 r/min for 15 min at 4°C.

Quantification of fecal SCFAs was performed as previously described using an Agilent 7890 A. Chromatographic conditions were as follows: injection volume 1 μL, inlet temperature 220°C, column flow 0.95 mL/min, column temperature 90°C, equilibrium time 0.5 min, temperature increased to 150°C by 5°C/min, retention time 9 min, detector temperature 230°C, hydrogen flow 40 mL/min, air flow 400 mL/min and tail blowing flow 40 mL/min.

The retention time of each chromatographic peak was used for qualitative analysis, and the peak areas of different concentrations of standards were used for quantification. The gas chromatogram of SCFA standards is shown in Supplementary Fig. 1, and standard curves are provided in Supplementary Table 2.

### Bile acid analysis by UPLC-TOF/MS

A 10 mg sample of small intestine contents was weighed and dissolved in 1,000 μL methanol (−20°C) before being vortexed for 60 sec and sonicated for 30 min. The supernatants were collected after centrifugation at 12,000 r/min for 10 min at 4°C, and then diluted 50-fold.

An ACQUITY UPLC ® BEH C18 column (2.1 × 100 mm, 1.7 μm, Waters Corp.) was used for chromatographic analysis. The injection volume was 5 μL, and the mobile phase solvents included 0.01% formic acid (A) and acetonitrile (B). Flow rate was 0.25 mL/min. Electrospray ionization (ESI) in positive and negative ion modes was used for Q-TOF mass spectrometric analysis. The ion source temperature was 500°C, with the voltage being 4,500 V. Nebulizing gas and auxiliary gas (nitrogen) were set at 50 psi, curtain gas was set at 30 psi, and collision gas was set at 6 psi. Lithocholic acid (LCA) and other substances were used as standards (Sigma, USA). The high-performance liquid chromatography (HPLC) chromatogram of BA standards is presented in Supplementary Fig. 2, and standard curves are provided in Supplementary Table 3.

### Gut microbiota analysis by 16S rRNA gene sequencing

DNA preparation, polymerase chain reaction (PCR) amplification, and pyrosequencing were performed as described by Li et al. (26). Briefly, total DNA was extracted from 250 mg of cecal contents using E.Z.N.A. Stool DNA kit (OMEGA, Bio-Tek, USA). A primer set (515F/926R) was used to amplify the V4 region of the 16S rRNA gene to analyze gut microbiota. Each sample was amplified in triplicate in a reaction volume of 20 μL containing 1 × Ex Taq PCR buffer, 10 pM each primer, 1.25 U Takara Ex Taq (TaKaRa Biotechnology Co., Ltd, Dalian, China), and 5 ng genomic DNA using the following program: 5 min at 95°C, followed by 30 cycles of 94°C for 30 sec, then 56°C for 30 sec, and 72°C for 30 sec, and finally, 10 min at 72°C. PCR products were purified using the QIAEX II Gel Extraction Kit (QIAGEN). Library preparation and pyrosequencing were performed at the Chinese National Human Genome Center in Shanghai on the 454 GS FLX sequencing platform (Roche). Raw sequences are available via the NCBI/EBI/DDBJ Sequence Read Archive (accession Nos. DRA002627 and DRA0012641).

The average read length was 250 bp. By randomly sampling 30,681 sequences from each sample, the resulting sequences could be rarefied separately, such that the sampling workload of the entire sample was equal. To predict the functional profiles of microbial communities, the operational taxonomic unit (OTU) was analyzed by clustering sequences at the 97% similarity level (USEARCH, version 10.0). OTUs were filtered based on a threshold set at 0.005% of all sequences. α-Diversity was represented by Shannon index at the OTU level, while β-diversity was represented by the Bray–Curtis (*BC*) distance. The results of principal component analysis (PCA) were plotted using R software.

### RNA isolation and quantification of gene expression

Total RNA was isolated from rat colon using Ribopure RNA extraction kit (Invitrogen, USA) by following the manufacturer’s instructions. cDNA synthesis was performed using 1 μg total RNA and a RT-PCR kit (Invitrogen, Rockville, MD) with a Super Script III First-Strand Synthesis System. cDNA synthesized from total RNA was evaluated using a real-time quantitative PCR system (CFX96 Touch™ Real-Time PCR Detection System, Bio-Rad, USA). Primers for genes targeted in this study are listed in Supplementary Table 4. Relative expression values were calculated using the 2-ΔΔCt method (27).

### Western blotting analysis

Western blot analysis was performed as described in the previous study (28). Proteins were separated on SDS-PAGE gels (8–12%), and then transferred onto Polyvinylidene difluoride (PVDF) membranes (Bio-Rad, CA). After being blocked with 5% dried skimmed milk, the membranes were incubated with primary antibodies overnight at 4°C, and then with horseradish peroxide-conjugated secondary antibodies. The resulting protein bands were visualized using a chemiluminescence system (FUSION, France), and densitometric analysis was performed using the ImageJ software (NIH, MD). The following primary antibodies were used: anti-CYP7A1, anti-farnesoid X receptor (anti-FXR) and anti-TRPV1 antibodies (Abcam, Waltham, MA).

### Statistical analysis

The homeostatic model assessment–insulin resistance (HOMA-IR) index was calculated via the formula: HOMA-IR = fasting insulin × fasting glucose/22.5 (29–31). All data are expressed as means ± standard deviations (*n* = 8). Data were subjected to one-way analysis of variance using Origin 8.5 and SPSS version 20.0. Differences among groups were examined using Duncan’s multiple-range test. *P* < 0.05 was considered to be statistically significant.

## Results

### CAP decreases weight gain, liver weight and food intake

**Fig. 1:**
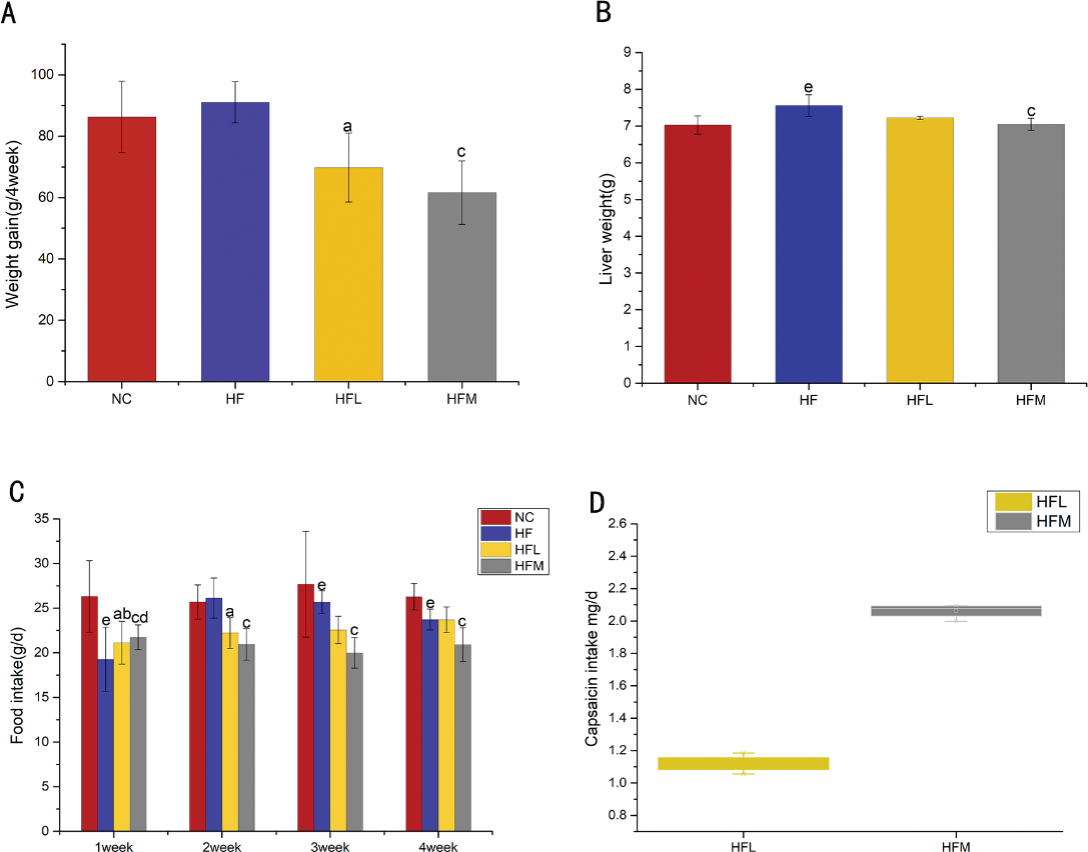
(A) Weight gain of SD rats fed with different doses of CAP and a high-fat diet. (B) Liver weight of SD rats fed with different doses of CAP and a high-fat diet. (C) Food intake of SD rats fed with different doses of CAP and a high-fat diet. (D) Intake of SD rats fed with different doses of CAP and a high-fat diet. Values are expressed as mean ± SD (*n* = 8). (a) HF group versus HFL group; (b) NC group versus HFL group; (c) HF group versus HFM group; (d) NC group versus HFM group; (e) NC group versus HF group (one-way ANOVA followed by Dunnett’s test, *P* < 0.05). NC group: control group; HF group: high-fat diet group; HFL group: high-fat diet treated with suboptimal dose of CAP; HFM group: high-fat diet treated with optimal dose of CAP.

The weight gain of SD rats fed different doses of CAP after 4 weeks is presented in Fig. 1A. There was no significant difference in weight gain between the NC and HF groups. Compared with the HF group, the weight gain was significantly decreased in the HFL and HFM groups. As shown in Fig. 1B, compared with the HF group, the liver weight was decreased the in the HFM group. As shown in Fig. 1C, the food intake of HFL and HFM groups from weeks 2 and 3 was significantly lower than that of the HF group. At week 4, the food intake of the HFM group was found to be 20.9 g/day, which was significantly lower than that of the HF group; however, the difference between the HFL and HF groups was not significant. Inferring *from the* differences in feed *intake of* the HFL and HFM groups, *we* calculated the consumption of capsaicin in the HFL group and the HFM group to be 1.12 mg/day and 2.06 mg/day, respectively (Fig. 1D).

### CAP improves dyslipidemia, hepatic lipid, fasting plasma glucose and serum fasting insulin

A high level of serum TC is considered a risk factor for atherosclerosis. Increased levels of HDL-C and decreased levels of TG and TC may reduce cardiovascular risk (32), The effects of different doses of CAP on blood lipids are shown in Table 1. Compared with the NC group, TG, TC, and HDL-C increased in the HF group; while compared with the HF group, HDL-C increased, TG, TC and hepatic lipids decreased in the HFL and HFM group, indicating that CAP may have cardiovascular benefits. Studies have suggested that obesity is associated with higher levels of FPG and fasting insulin (33, 34). Hence, we performed a comparative analysis of the FPG and fasting insulin, and we found that compared with the HF group, levels of FPG and fasting insulin were decreased in the HFL and HFM groups. Moreover, HFL had a better regulatory effect on fasting insulin. HOMA-IR is a homeostatic model assessment (HOMA) to determine insulin resistance (IR) in β-cells (31). Compared with the NC group, HOMA-IR was increased in HF. Compared with the HF group, HOMA-IR was decreased in the HFL and HFM group.

**Table 1:** Effects of capsaicin on plasma parameters in SD rats

Plasma parameters	NC	HF	HFL	HFM
TC (mmol/L)	3.07 ± 0.353	4.07 ± 0.764^e^	3.20 ± 0.049^a^	3.24 ± 0.212^c^
TG (mmol/L)	0.46 ± 0.007	0.71 ± 0.163^e^	0.53 ± 0.064^a^	0.50 ± 0.141^c^
LDL-C (mmol/L)	1.29 ± 0.297	1.61 ± 0.028	1.48 ± 0.156	1.52 ± 0.488
HDL-C (mmol/L)	1.28 ± 0.113	0.96 ± 0.028^e^	1.17 ± 0.007^a^	1.23 ± 0.007^c^
Fasting insulin (mU/L)	50.26 ± 5.742	61.03 ± 1.018^e^	45.550.255^a^	51.40 ± 2.673^c^
Fasting plasma glucose (mmol/L)	4.82 ± 0.064	5.30 ± 0.403	4.51 ± 0.247^a^	4.07 ± 0.177^c^
Hepatic lipids (mg/g)	31.48 ± 1.724	38.72 ± 1.598^e^	34.647 ± 2.177^a^	33.29 ± 1.098^c^
HOMA-IR	10.76 ± 0.405	14.36 ± 0.854^e^	9.12 ± 0.450^a^	9.30 ± 0.887^c^

Note: Data are expressed as mean ± SD (*n* = 8). Values are mean ± SD (*n* = 8).

a HF group versus HFL group.

b NC group versus HFL group.

c HF group versus HFM group.

^d^NC group versus HFM group.

e NC group versus HF group (one-way ANOVA followed by Dunnett’s test, *P* < 0.05).

### CAP decreases inflammatory factors in HFD-fed rat

**Fig. 2:**
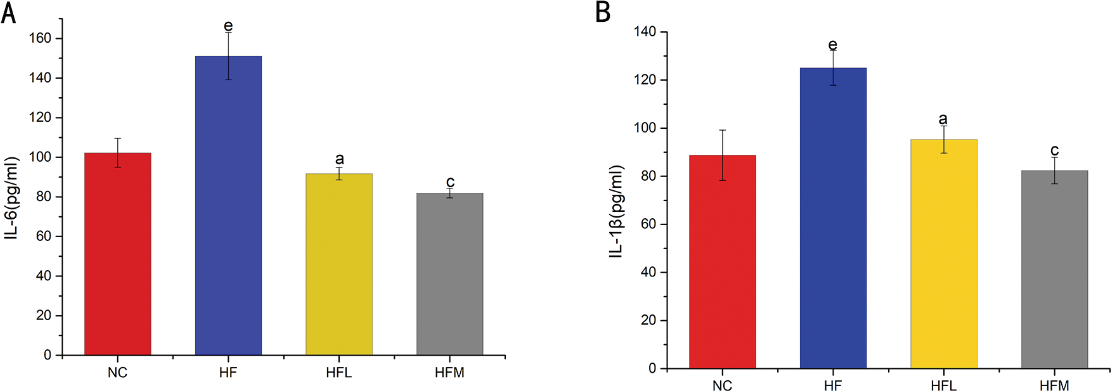
(A, B). Levels of plasma inflammatory factors for different doses of CAP in SD rats fed a high-fat diet. (A) Concentrations of serum IL-6. (B) Concentrations of serum IL-1β. Values are expressed as mean ± SD (*n* = 8). (a) HF group versus HFL group; (b) NC group versus HFL group; (c) HF group versus HFM group; (d) NC group versus HFM group; (e) NC group versus HF group (one-way ANOVA followed by Dunnett’s test, *P* < 0.05). NC group: control group; HF group: high-fat diet group; HFL group: high-fat diet treated with suboptimal dose of CAP; HFM group: high-fat diet treated with optimal dose of CAP.

Many studies have shown that pro-inflammatory factors increase in rats fed a HFD (35, 36). Overproduction of interleukin-6 (IL-6) and interleukin-1β (IL-1β) is an important feature of obesity, which contributes significantly to IR (37, 38). Therefore, we measured plasma levels of IL-6 and IL-1β across the four groups of rats. As shown in Fig. 2A and B, IL-6 levels were significantly increased in the HF group (by 63.6%) compared with that in the NC group, while IL-6 levels were decreased in HFL (by 73.6%) and HFM (by 80%) groups compared with that in the HF group. The IL-1β levels in the HFM (by 34.2%) and HFL (by 23.8%) groups were significantly decreased compared with that in the HF group (125.07 pg/mL).

### CAP improves the diversity and structure of gut microbiota

**Fig. 3:**
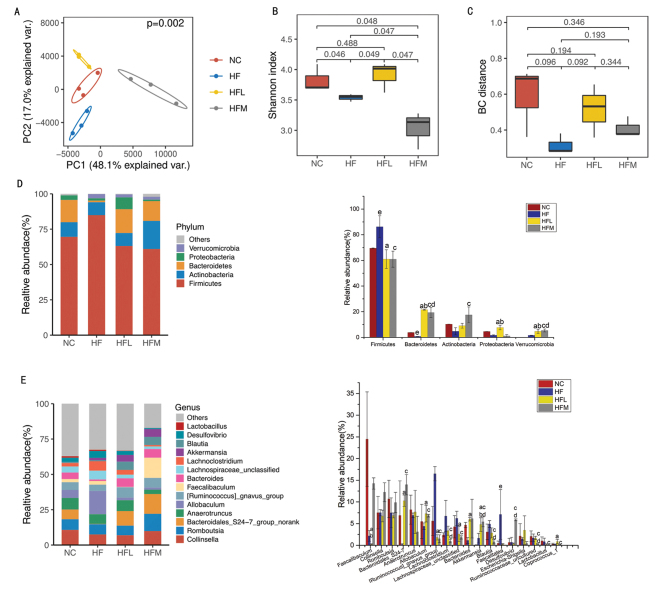
(A) Principal component analysis (PCA) at OTU level; (B) Shannon index at OTU level. (C) Bray–Curtis distance at OTU level. (D) Phylum-level bar plot and differences in relative abundance of microorganisms at the phylum level. (E) Genus-level bar plot and differences in relative abundance of the first 20 species of microorganisms. (a) HF group versus HFL group; (b) NC group versus HFL group; (c) HF group versus HFM group; (d) NC group versus HFM group; (e) NC group versus HF group (one-way ANOVA followed by Dunnett’s test, *P* < 0.05). NC group: control group; HF group: high-fat diet group; HFL group: high-fat diet treated with suboptimal dose of CAP; HFM group: high-fat diet treated with optimal dose of CAP.

To understand the effects of CAP on intestinal microflora in SD rats, we performed PCA based on the OTU. From PCA, a PCA diagram was drawn, and similarities and differences between samples were evaluated (Fig. 3A). Based on this figure, we found that rats treated with the optimal dose of CAP were significantly different from those in the NC and HF groups, while rats treated with a suboptimal dose of CAP were significantly different from those in the HF group. These results revealed that the administration of CAP for 4 weeks induced obvious changes to the intestinal microbial communities of HFD SD rats, and thus, reducing the impact of HFD. Figure 3B illustrates that the Shannon index of CAP treatment had a significant effect on the α-diversity of gut microbiota in rats fed an HFD. Furthermore, the Bray–Curtis (BC) distance revealed slightly significant differences between the HFL and HF groups (Fig. 3C). Results of both α- and β-diversity analyses indicated that the intestinal microflora of SD rats treated with CAP were significantly different from those of rats under HF treatment, while intestinal microflora in the groups receiving a suboptimal dose of CAP were similar to those in the NC group.

The structures of intestinal flora at the level of phylum are shown in Fig. 3D, and the top five abundant phyla of flora were *Firmicutes*, *Verrucomicrobia, Bacteroidetes*, *Actinobacteria*, and *Proteobacteria.* Compared with HF groups, *Ruminococcus_gnavus_group* and *Lachnospiraceae* decreased in HFL and HFM groups, while *Bacteroidales_S24-7, Akkermansia, Allobaculum*, and *Coprococcus* increased in HFL and HFM groups. Compared with HF group, *Blautia* and *Lactobacillus* decreased in HFM group but *Desulfovibrio* increased in HFM group, as shown in Fig. 3E

### CAP affects composition of BAs

**Fig. 4:**
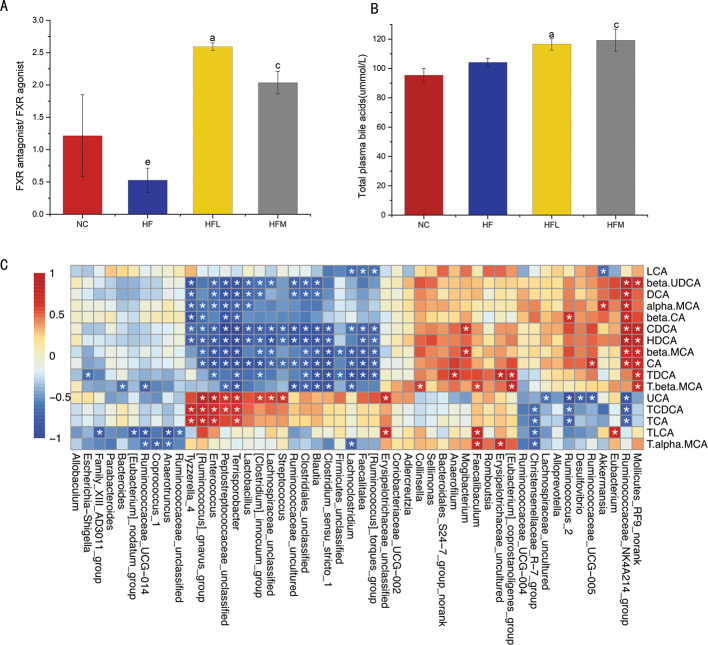
(A) FXR antagonist/agonist BAs in the intestinal microflora were calculated. (B) Total concentration of BAs in plasma. Values are expressed as mean ± SD (*n* = 8). (a) HF group versus HFL group; (b) NC group versus HFL group; (c) HF group versus HFM group; (d) NC group versus HFM group; (e) NC group versus HF group (one-way ANOVA followed by Dunnett’s test, *P* < 0.05). NC group: control group; HF group: High fat diet group; HFL group: high-fat diet treated with suboptimal dose of CAP; HFM group: high-fat diet treated with optimal dose of CAP. (C) Pearson correlation heatmap between key gut microbial taxa and BA profiles. Red color denotes a positive association, blue denotes a negative association, and white denotes no association. Data are expressed as mean ± SD. One-way ANOVA with Bonferroni post hoc test was used for data analysis. *P < 0.05.

BAs, derived from cholesterol in the liver, are an important compound in the gut lumen produced in response to ingestion of dietary fat (39, 40). In this study, 15 BA species were analyzed to assess the changes that occurred due to remodeling of the gut microbiota following CAP treatment (Table 2). Compared with the NC group, levels of α-MCA, LCA, and taurodeoxycholic acid (TDCA) decreased but taurolithocholic acid sodium salt (TLCA) increased in the HF group, suggesting that HFD altered the composition of BAs. Compared with the HF group, levels of most unconjugated BAs, including chenodeoxycholic acid (CDCA), β-MCA, α-MCA, and deoxycholic acid (DCA), were increased in HFL and HFM groups (Table 2); levels of conjugated BA taurochenodeoxycholic acid (TCDCA) and TLCA were decreased, while TDCA sodium salt and Tauro-β-muricholic acid (T-β-MCA, sodium salt) were significantly increased in HFL and HFM groups compared with the HF group (Table 2). The administration of CAP yielded a BA pool with increased FXR antagonistic BAs and decreased FXR agonistic BAs compared with the pool in the HF group (Fig. 4A). Figure 4B shows the concentration of BAs in plasma, HFL and HFM significantly higher than the HF and NC group.

**Table 2:** Effects of capsaicin on bile acid analysis

Bile acids	NC	HF	HFL	HFM
Unconjugated BA				
LCA (ug/mL)	58.33 ± 3.21	15.58 ± 1.06^e^	24.29 ± 3.31	34.55 ± 8.08^c,d^
β-UDCA (ug/mL)	4.41 ± 4.19	3.24 ± 0.98	7.15 ± 2.69	27.71 ± 21.04
α-MCA (ug/mL)	14.53 ± 1.26	47.19 ± 5.70^e^	103.00 ± 21.47^a,b^	110.23 ± 14.70^c,d^
β-MCA (ug/mL)	318.76 ± 26.39	263.96 ± 15.64	472.16 ± 58.05^a,b^	704.57 ± 51.92^c,d^
DCA (ug/mL)	82.78 ± 12.11	146.80 ± 1.50	772.35 ± 91.36^a,b^	980.13 ± 105.55^c,d^
CDCA (μg/mL)	167.51 ± 33.02	163.45 ± 0.84	360.72 ± 23.74^a,b^	636.01 ± 32.75^c,d^
HDCA (μg/mL)	95.03 ± 110.22	90.60 ± 65.85	157.49 ± 2.39	303.00 ± 186.81
UCA (μg/mL)	1462.97 ± 1570.11	155.055 ± 939.36	522.96 ± 102.03	1113.03 ± 459.96
CA (μg/mL)	4402.56 ± 4559.56	3766.91 ± 1574.26	5016.76 ± 616.38	8589.39 ± 907.83
Conjugated BA				
TLCA (μg/mL)	17.00 ± 3.46	28.37 ± 7.58	6.54 ± 0.47^a,b^	26.23 ± 5.71
TDCA (μg/mL)	328.72 ± 47.52	120.25 ± 3.53^e^	228.87 ± 34.63^a,b^	439.78 ± 39.75^c,d^
TCDCA (ug/mL)	1013.79 ± 156.75	467.42 ± 79.38^e^	119.73 ± 16.27^a,b^	274.07 ± 5.99^c,d^
T-α-MCA (μg/mL)	1952.05 ± 193.27	1455.40 ± 150.15	654.62 ± 32.77	2017.31 ± 340.92
T-β-MCA (μg/mL)	3754.73 ± 3128.32	1642.10 ± 834.48	17118.76 ± 1879.05^a,b^	21902.83 ± 114.78^c,d^
TCA (μg/mL)	12562.31 ± 1826.23	6051.37 ± 1325.84^e^	2420.18 ± 78.34^a,b^	4192.24 ± 281.98^d^

Note: Data are expressed as mean ± SD (*n* = 8). Values are expressed as mean ± SD (*n* = 8).

a HF group versus HFL group.

b NC group versus HFL group.

c HF group versus HFM group.

d NC group versus HFM group;

e , NC group versus HF group (one-way ANOVA followed by Dunnett’s test, *P* < 0.05).

Pearson correlation heatmap analysis showed a link between gut microbiota genera and BA profiles (Fig. 4B). It was observed that *Desulfovibrio* was negatively correlated with Ursocholic acid (UCA), while *Akkermansia* was negatively correlated with FXR agonist LCA and positively associated with α-MCA. *Bacteroides* was negatively correlated with T-β-MCA. Some studies have shown that *Lactobacillus* is negatively correlated with FXR antagonists (T-β-MCA) and positively associated with FXR agonists, such as taurocholic acid sodium salt (TCA) (28). The study results are consistent with this finding; *Lactobacillus* levels were negatively correlated with T-β-MCA and positively associated with TCA. While these differences did not reach significant levels, this may be due to limitations of our small sample size.

### CAP Improves dyslipidemia through the FXR signaling pathway

**Fig. 5:**
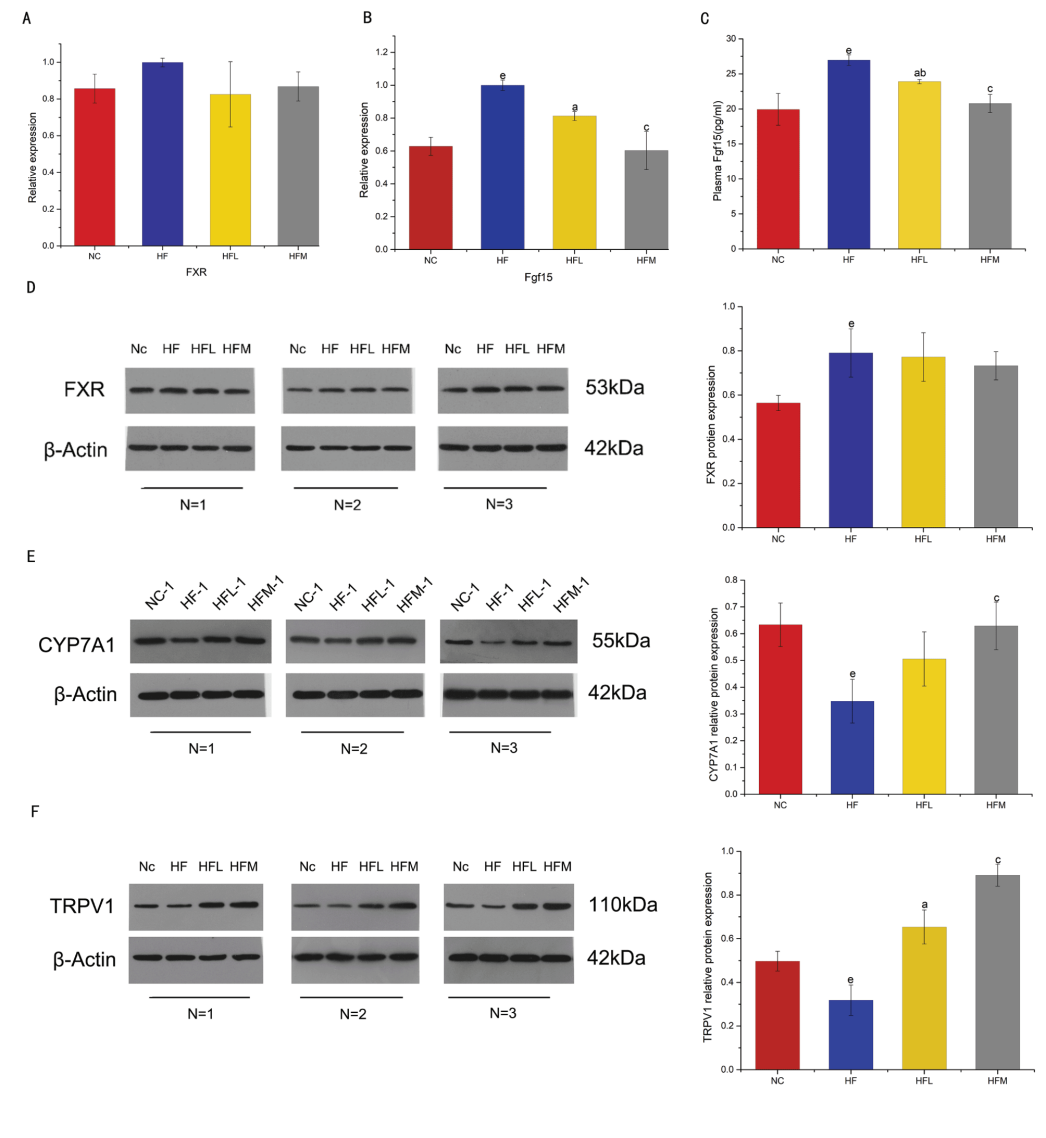
(A, B) FXR and Fgf15 mRNA expression in the colon of SD rats fed a high-fat diet was measured using qRT-PCR to explore the effect of different doses of CAP. (C) The concentration of Fgf15 in plasma to explore the effect of different doses of CAP. (D) Western blotting was used to detect FXR expression in the liver of SD rats and the relative protein expression levels were quantified by densitometry. (E) Western blotting was used to detect CYP7A1 expression in the liver of SD rats, and the relative protein expression levels were quantified by densitometry. (F) Western blotting was used to detect TRPV1 expression in the liver of SD rats, and the relative protein expression levels were quantified by densitometry. Values are expressed as mean ± SD (*n* = 8). (a) HF group versus HFL group; (b) NC group versus HFL group; (c) HF group versus HFM group; (d) NC group versus HFM group; (e) NC group versus HF group (one-way ANOVA followed by Dunnett’s test, *P* < 0.05). NC group: control group; HF group: high-fat diet group; HFL group: high-fat diet treated with suboptimal dose of CAP; HFM group: high-fat diet treated with optimal dose of CAP.

The alteration of intestinal BA profile may affect BA receptors, we next detected the expression of typical BA receptors FXR in colon of the rats. The relative mRNA expression of FXR did not exhibit statistical difference among groups but the express level in the HFL and HFM groups were slightly lower than that of HF group (Fig. 5A). We then detected the relative mRNA expression of FGF15 in colon and concentration of Fgf15 in plasma, which is closely related to FXR activity (Fig. 5B,C). We found that CAP intervention reversed the relative mRNA expression of colonic FGF15 and concentration of Fgf15 in plasma in rats, even in the HFL group. We also detected liver FXR, CYP7A1 and TRPV1 (Fig. 5D–F), and found CYP7A1 increased in HFM (by 80.9%), TRPV1 increased in HFM (by 179.7%), and the increased CYP7A1 activity may result in decreased intrahepatic cholesterol concentration (41, 42). TRPV1 is a specific receptor for CAP, Therefore, the expression of protein TRPV1 was also determined, and we found protein TRPV1 increased in the CAP group. This result is consistent with those reports by Ferdowsi and Zhang lei et al. (22, 43). Although not significant, protein expression of FXR in HFL and HFM slightly decreased when compared with HF, the similar results were reported in the literature of Hui et al. (28).

### CAP promotes the accumulation of SCFAs

SCFAs are the end products of dietary fibers through microbial fermentation, and they play an important role in the prevention and treatment of obesity (44). The concentrations of SCFAs in the cecal digesta of the experimental rats are shown in Table 3. Compared with the HF group, acetic acid, propionic acid, and i-butyric acid were significantly increased in HFL and HFM groups. Although butyric acid was increased in HFL (5.75 mmol/L) and HFM (5.72 mmol/L) groups, the difference was not significant. The content of total SCFAs in the HFL group was close to that of the NC group.

**Table 3:** Effects of CAP on fecal SCFA excretion

SCFAs	NC	HF	HFL	HFM
Acetic acid	13.88 ± 0.11	13.99 ± 1.03	15.75 ± 0.35^a,b^	15.19 ± 0.02^c,d^
Propionic acid	10.55 ± 0.09	9.91 ± 0.404^e^	10.69 ± 0.228^a^	10.49 ± 0.001^c^
Butyric acid	5.86 ± 0.242	5.53 ± 0.001^e^	5.75 ± 0.036	5.72 ± 0.002
i-Butyric acid	7.88 ± 0.135	7.59 ± 0.261	8.06 ± 0.168^a^	7.97 ± 0.025^c^
i-Valeric acid	4.96 ± 0.022	4.64 ± 0.001	5.05 ± 0.101	4.89 ± 0.024
n-Valeric acid	4.46 ± 0.027	ND	4.51 ± 0.057	4.51 ± 0.004
Total	45.82 ± 5.338	38.27 ± 4.479	49.81 ± 6.633^a^	48.84 ± 0.095^c^

Note: Data are expressed as mean ± SD (*n* = 8). Values are mean ± SD (*n* = 8). ND denotes not detected.

a HF group versus HFL group.

b NC group versus HFL group.

c HF group versus HFM group.

d NC group versus HFM group.

e NC group versus HF group (one-way ANOVA followed by Dunnett’s test, *P* < 0.05).

## Discussion

In this study, we validated the associations between gut microbiota and metabolites by adding different doses of CAP to an HFD. The study results showed 1) CAP changes the composition of BAs in the intestines and inhibits fat accumulation; 2) the presence of specific fermentative bacteria populations in high-fat-CAP diet; 3) less than optimal dose can also reverse lipid metabolism abnormalities and reduce weight without increasing abundance of *Desulfovibrio*.

In this study, the weight gain was decreased in the CAP-treated group, compared with other groups, consistent with previous studies (13, 25). There was no significant difference in weight gain between the NC and HF groups, consistent with Northcott’s finding that weight gain was not significantly increased for the first 10 weeks (25, 45). Food intake was significantly reduced in optimal-dose CAP group, which may be because the taste of CAP stimulates the intake of rats. This finding demonstrated that CAP can depress appetite and reduce feed intake, consistent with the reports of van Avesaat and Yuanwei Wang et al., which showed that the addition of dietary CAP may increase satiety and reduce appetite by enhancing glucagon-like peptide-1 (GLP-1) and peptide YY (PYY) expression (13, 46). After the analysis of blood biochemical indexes, we found that CAP can alleviate dyslipidemia and IR in the case of HFD, and even a suboptimal dose can significantly reduce blood lipid levels. Our results are consistent with those of Zhang Lei and Zhang Shiqi et al. (22, 47).

Gut microbiomes actively participate in the regulation of host metabolism and are mutualist partners of the host. It has been well-recognized that microbial localization, diversity, and composition vary across different parts of the gastrointestinal tract. In the cecum, the dominant microflora include *Bacteroidetes* and *Firmicutes*, and the *Firmicutes/Bacteroidetes* (F/B) ratio is modulated by dietary components (1). Many studies have shown that an increased ratio of *Firmicutes* to *Bacteroides* is associated with obesity (48). *Firmicutes* can inhibit the release of lipoprotein lipase (LPL) inhibitor, increase LPL activity, and encourage the accumulation of excess energy into fat (49). A significantly higher proportion of *Firmicutes* and a lower proportion of *Bacteroidetes* have been identified in obese animals compared with lean ones (49). This phenomenon was also confirmed in this study (Fig. 3D). The abundance of *Firmicutes* in SD rats was increased in the HFD group; however, it was decreased in the HFL and HFM groups treated with CAP. The reduced abundance of *Firmicutes* in rats treated with CAP indicates that CAP may help to reverse the intestinal microbial abnormalities caused by an HFD.

B*acteroidales_*S24-7 can degrade complex polysaccharides into acetate, propionate, and succinate (50). Furthermore, B*acteroidales_*S24-7 may be involved in mediating the effects of treatments for glucose intolerance and obesity (13). Propionate and acetate make up specific SCFAs, and the protective role of SCFAs against gut inflammation has been well demonstrated (51). In this study, *Bacteroidales_*S24-7 was increased at the genus level in HFL and HFM rats (Fig. 3E), suggesting the potential therapeutic value of CAP in treating the imbalance of intestinal microflora induced by HFD.

*Akkermansia* is a mucin-degrading bacterium with both regulatory and inflammatory properties (52). Many microbiome studies based on other natural foods, such as black tea, polyphenols, bamboo shoot fiber, and cranberry, have suggested that *Akkermansia* might mediate these anti-obesity effects (53, 54). With regard to CAP, Ritesh et al. used qPCR to detect differences in the abundances of seven gut microbes and found that the relative abundance of *Akkermansia* was higher in HFD + CAP-fed mice (14). In agreement with these findings, we observed that the relative abundance of *Akkermansia* was increased in the HFL and HFM groups (Fig. 3E).

In this study, the optimal dose of CAP increased the abundance of *Desulfovibrio,* and some research studies reported that the increased abundance of *Desulfovibrio* might be an indicator of colitis development (55); thus, optimal-dose CAP treatment may increase the risk of colitis in SD rats. *Desulfovibrio* has been reported to be negatively correlated with levels of LDL-C, TG, and TC (56), which was also observed in this study.

*Lactobacillus* is a bile salt hydrolase (BSH) producing microbiome, where BSH is critically involved in the hydrolysis of conjugated BAs by gut microbiota (57). At the genus level, CAP administration prevented the increase in abundance of the genus *Lactobacillus* in SD rats, especially in the optimal dose group, the result of which was consistent with the report of Hui et al. (28). This change means that BSH activity was reduced, leading to increased levels of conjugated BA, especially T-β-MCA (28). In this study, we also observed that CAP administration enhanced the accumulation of T-β-MCA. The decrease of *Lactobacillus* abundance could lead to changing the intestinal environment dramatically, such as pH, and then the abundance of harmful bacteria appeared to increase (58). In this study, the genera abundances of *Desulfovibrio* were significantly increased in the CAP group*.*

In this study, CAP generally increased abundances of *Bacteroidales_S24-7*, *Akkermansia, Allobaculum,* and *Coprococcus*, which is consistent with the reports of Wang Yuanwei (13) and Shen et al. (15). However, *Desulfovibrio* was not observed to decrease in the CAP group in this study. Instead, we found that *Desulfovibrio* increased in the optimal dose group. CAP treatment also decreased abundances of *Ruminococcus_gnavus_group, lactobacillus*, and *Faecalitalea* (Fig. 3E). These results suggest that CAP can reduce weight and dyslipidemia by changing the population structure of gut microbiota and promoting the accumulation of SCFAs.

SCFAs are the final products of dietary fibers and resistant starch through microbial fermentation, and they play an important role in the prevention and treatment of obesity (44). This research study suggest that the less or optimal dose of CAP may facilitate the production of SCFAs, acetic and propionic acids from intestinal microbiota, thus indicating that CAP improved lipid accumulation thereby decreasing serum TG and TC levels. Butyric acid plays important roles in regulating growth performance, gastrointestinal function, immunity, gastrointestinal microecological balance, and intestinal pH, and it also has bactericidal and bacteriostatic functions (59). In this research study, butyric acid was slightly increased in less and optimal dose of CAP, although the difference was not remarkable. This result suggest that CAP can regulate gastrointestinal microecological balance.

As BAs are potent ‘digestive surfactants’, through the activation of various signaling pathways, BAs can not only regulate their own synthesis and enterohepatic circulation, but also contribute to the maintenance of TG, cholesterol, glucose, and energy homeostasis (60). In addition, intestinal bacteria can combine BAs with cholesterol molecules to excrete BA–cholesterol complexes in feces (56). The FXR is a key regulator of BA metabolism, which can also influence glucose and lipid metabolism (61). The FXR regulates the transcription of many hepatic and intestinal genes, including mouse fibroblast growth factor (FGF) 15 and its human homologue, FGF19, to maintain cholesterol-BA homeostasis, reduce hepatic gluconeogenesis, and suppress inflammation (62). In this study, CAP-fed rats showed significantly increased levels of most unconjugated BAs, including DCA, CDCA, β-MCA, and α-MCA.

CDCA, DCA, LCA, CA, and β-MCA are the most potent ligands of FXR (63). In this study, the levels of CDCA, DCA, T-β-MCA, and β-MCA were increased after administration of CAP, which led to change in FXR. The activation of intestinal FXR has been reported to improve metabolic disorders in db/db mice (64). However, other studies reported that intestinal FXR antagonist was beneficial for treating obesity in mice (65). Thus, intestinal FXR might exert bidirectional regulation of metabolic diseases. In this study, CAP significantly increased the accumulation of conjugated BAs, especially T-β-MCA. T-β-MCA is a natural FXR antagonist and can increase BA synthesis, leading to a larger BA pool through the repression of the enterohepatic FXR-FGF15 axis (66). This antagonism can improve metabolism via reducing biosynthesis of intestinal-derived ceramides (67). Ceramides are mediators of cell death, inflammation, and IR (68). In addition, in this study, we found a change in the ratio of antagonistic/agonistic ligands for FXR, which likely affected enterohepatic FXR signaling. Therefore, the repression of enterohepatic FXR-FGF15 signaling likely occurred due to the alteration of BA profiles, which promoted CYP7A1 expression and hepatic BA synthesis. This was supported by our findings that FGF15 decreased while CYP7A1 increased in the CAP group. Elevated levels of lCYP7A1 promotes the conversion of cholesterol to cholic acid, resulting in BA secretion and nutrient absorption (69). A previous research study showed that overexpression of CYP7A1 in the liver led to increased hepatic cholesterol catabolism and BA pool, while decreased the plasma cholesterol content (70). In this study, we also observed increased levels of CYP7A1, total BAs, and decreased levels of TG and TC in the CAP group. The reason for an increased circulating level of total BAs in the blood and lowering of hepatic cholesterol may be due to a subsequent increase in the conversion of cholesterol to BAs by the liver (71).

In this study, increased expression of TRPV1 caused by CAP administration can further lead to increased intracellular calcium levels and several related cellular responses (72). Increased levels of cytosolic calcium can activate phosphorylation of AMPK, thereby regulating glucose and fat metabolism (43). This might be a reason why CAP can improve fasting glucose and insulin levels.

## Conclusions

Overwhelming evidence has emphasized the importance of gut microbiota in metabolic diseases affecting key pathways, such as energy homeostasis and inflammation. By investigating intestinal microorganisms, intestinal BAs, and SCFAs, this study found that CAP treatment can reduce body weight and ameliorate the abnormal lipid and glucose metabolism response caused by consumption of an HFD. Specifically, we found that CAP administration regulates BA composition thus activating FXR, which, in turn, inhibits the expression of Fgf15 to promote expression of protein CYP7A1, thereby regulating lipid metabolism and lowering TG and TC levels. Furthermore, CAP induces remodeling of the gut microbiota, characterized by increased abundance of *Bacteroidales S24-7* group, *Allobaculum,* and *Akkermansia*, thereby leading to accumulation of SCFAs that protects intestinal health, and inhibiting lipogenesis to maintain intestinal homeostasis. However, in this study, the use of optimal dose of CAP enhanced the regulation of glycolipid metabolism; however, it increased the abundance of *Desulfovibrio*, which can increase the risk of colon cancer. Therefore, the less optimal dose of CAP can be used to improve metabolic abnormalities.

## Authors’ contributions

Ting Gong performed main experiments. Ting Gong, Min Zhang, and Xiong Liu conceived and designed research. Haizhu Wang, Shanli Liu, and Yong Xie participated in the discussion of the experiment and the review of the study. Ting Gong wrote the first draft of the article. All authors contributed to discussing the results, editing the article, and approved the final version of the manuscript.

## Conflict of interest and funding

All authors declare that they have no conflict of interest. The authors have not received any funding or benefits from industry or elsewhere to conduct this study

## Ethics statement

All procedures followed were in accordance with the ethical standards of the responsible committee on human experimentation Institutional Animal Care and Use Committee of Southwest University.

## Supplementary Material

Capsaicin regulates lipid metabolism through modulation of bile acid/gut microbiota metabolism in high-fat-fed SD rats
