# Supplementary material for: Capsaicin regulates lipid metabolism through modulation of bile acid/gut microbiota metabolism in high-fat-fed SD rats
Source: Food Nutr Res. 2022 May 26;66:10.29219/fnr.v66.8289. doi: 10.29219/fnr.v66.8289 (PMC9180124; doi:10.29219/fnr.v66.8289)
Supplement: Capsaicin regulates lipid metabolism through modulation of bile acid/gut microbiota metabolism in high-fat-fed SD rats [file FNR-66-8289-s001.docx]

**Figure Captions**

**Fig. S1.** Gas chromatogram of short-chain fatty acids in standard solution (20 mmol/L).

**Fig. S2.** HPLC chromatogram of bile acids (BAs) in standard solution.

**Figure S1**


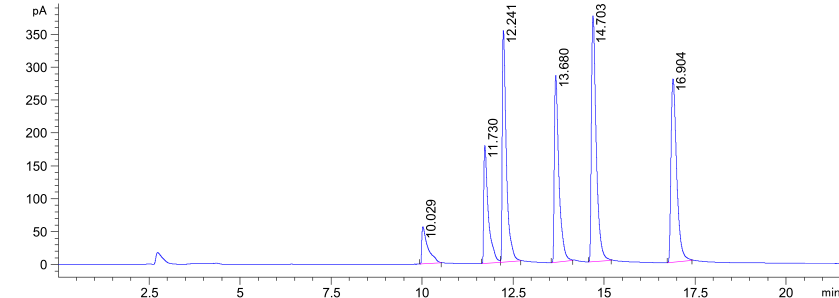


**Figure S2
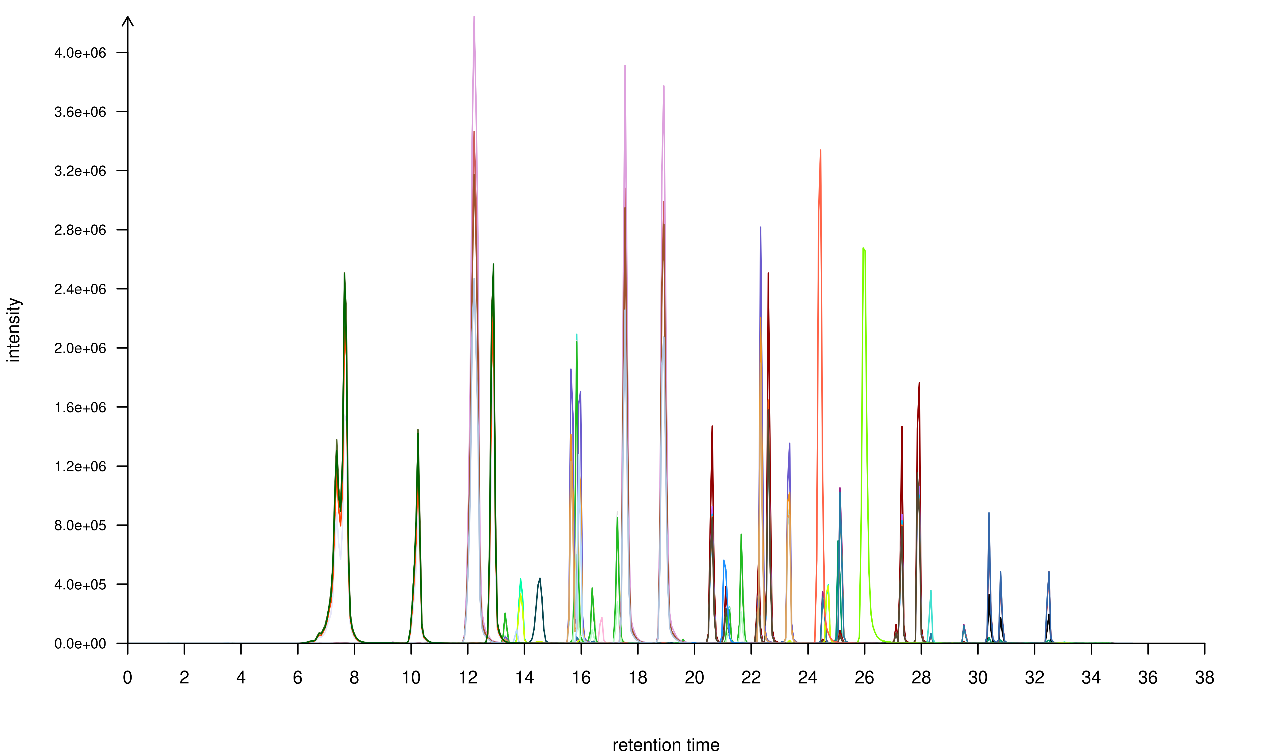
**

**Table S1** The ingredient compositions of the experimental diets (g/kg Diet)

| Ingredients | NC | HF |
| --- | --- | --- |
| Corn starch | 549.5 | 449.5 |
| Lard | 0 | 100 |
| Soybean oil | 40 | 40 |
| a AIN-76 mineral mixture (%mixture) | 35 | 35 |
| Casein | 200 | 200 |
| b AIN-76 vitamin mixture (%mixture) | 10 | 10 |
| Sucrose | 100 | 100 |
| L-cystine | 3 | 3 |
| Cellulose | 50 | 50 |
| Choline Chloride | 2.5 | 2.5 |
| Cholesterol | 10 | 10 |

**Table S2** Calibration curves of different short-chain fatty acids (SCFAs) in standard solution

| SCFAs | Linear equation | Correlation coefficient (R^2^) |
| --- | --- | --- |
| Acetic acid | y=62.583x-795.68 | 0.9914 |
| Propionic acid | y=121.56x-1144.4 | 0.9963 |
| i-butyric acid | y=171.47x-942.01 | 0.9988 |
| n-butyric acid | y=171.6x-1250.1 | 0.998 |
| i-valeric acid | y=193.82x-893.63 | 0.9996 |
| n-valeric acid | y=204.8x-843.75 | 0.9997 |

**Table S3** Calibration curves of different Bile acids (BAs) in standard solution

| Bile acid | Full name | Retention time（min） | linear equation | Correlation coefficien（R） |
| --- | --- | --- | --- | --- |
| LCA | Lithocholic acid | 32.49 | Y=-1219.6 + 2452.7*X | 0.996509278 |
| β-UDCA | 3β-Ursodeoxycholic acid | 20.61 | Y=-826.9 + 8195.7*X | 0.997482682 |
| DCA | Deoxycholic acid | 27.88 | Y=3170.5 + 6339.8*X | 0.998563291 |
| CDCA | Chenodeoxycholic acid | 27.3 | Y=-1829.4 + 3711.6*X | 0.995607743 |
| HDCA | Hyodeoxycholic acid | 22.6 | Y=299.04 + 3227*X | 0.997026219 |
| α-MCA | α-Muricholic acid | 15.84 | Y=-108.95 + 4376.1*X | 0.995819998 |
| UCA | Ursocholic acid | 13.32 | Y=-447.66 + 1843.2*X | 0.997831515 |
| β-MCA | β-Muricholic acid | 17.28 | Y=132.52 + 1879.9*X | 0.996643952 |
| CA | Cholic acid | 21.65 | Y=1176.8 + 2948.6*X | 0.999327695 |
| TLCA | Taurolithocholic acid Sodium Salt | 24.44 | Y=-496.67 + 12631*X | 0.998144375 |
| TDCA | Taurodeoxycholic acid Sodium Salt | 18.92 | Y=285.19 + 9813*X | 0.99745871 |
| TCDCA | Taurochenodeoxycholic acid | 17.56 | Y=-296.28 + 8556.7*X | 0.998502153 |
| TCA | Taurocholic acid Sodium Salt | 12.96 | Y=-68.605 + 4988.1*X | 0.999233726 |
| T-α-MCA | Tauro-α-muricholic acid Sodium Salt | 7.391 | Y=305.65 + 8644.9*X | 0.995792518 |
| T-β-MCA | Tauro-β-muricholic acid Sodium Salt | 7.662 | Y=27.883 + 7597*X | 0.99666197 |

**Table S4** Sequences of primers used for RT-qPCR

| **Gene** | **Forward** | **Reverse** |
| --- | --- | --- |
| β-actin | GACCCAGATCATGTTTGAGA | GCAGTAATCTCCTTCTGCATCC |
| Fxr | GTGACAAAGAAGCCGCGAAT | GCAGGGTGAGCGCGTTGTAAT |
| Fgf15 | AAGTGGAGTGGGCGTATTGT | AGTGGACCTTCATCCGACAC |
